# Hypoxia-inducible factor and cellular senescence in pulmonary aging and disease

**DOI:** 10.1007/s10522-025-10208-z

**Published:** 2025-02-26

**Authors:** Riya Thapa, Arockia Babu Marianesan, A. Rekha, Subbulakshmi Ganesan, Mukesh Kumari, Asif Ahmad Bhat, Haider Ali, Sachin Kumar Singh, Amlan Chakraborty, Ronan MacLoughlin, Gaurav Gupta, Kamal Dua

**Affiliations:** 1 Uttaranchal Institute of Pharmaceutical Sciences, Uttaranchal University, Dehradun, India; 2 School of Pharmaceutical Sciences, GLA University, Mathura, India; 3 Dr D Y Patil Medical College, Hospital and Research Centre, Pimpri, Pune, India; 4 Department of Chemistry and Biochemistry, School of Sciences, JAIN (Deemed to Be University), Bangalore, Karnataka India; 5 NIMS Institute of Engineering & Technology, NIMS University Rajasthan, Jaipur, India; 6 Centre for Global Health Research, Saveetha Medical College, Saveetha Institute of Medical and Technical Sciences, Saveetha University, Chennai, India; 7 School of Pharmaceutical Sciences, Lovely Professional University, Phagwara, Punjab India; 8 Faculty of Health, Australian Research Centre in Complementary and Integrative Medicine, University of Technology, Sydney, Ultimo, NSW 2007 Australia; 9 Division of Immunology, Immunity to Infection and Respiratory Medicine, Faculty of Biology, Medicine and Health, The University of Manchester, Oxford Road, Manchester, M13 9PL UK; 10 Aerogen, IDA Business Park, Dangan, Galway, H91 HE94 Ireland; 11 School of Pharmacy & Biomolecular Sciences, Royal College of Surgeons in Ireland, Dublin, D02 YN77 Ireland; 12 School of Pharmacy & Pharmaceutical Sciences, Trinity College, Dublin, D02 PN40 Ireland; 13 Centre for Research Impact & Outcome, Chitkara College of Pharmacy, Chitkara University, Rajpura, Punjab, 140401 India; 14 Centre of Medical and Bio-Allied Health Sciences Research, Ajman University, Ajman, United Arab Emirates; 15 Discipline of Pharmacy, Graduate School of Health, University of Technology, Sydney, Ultimo, NSW 2007 Australia; 16 Woolcock Institute of Medical Research, Macquarie University, Sydney, Australia

**Keywords:** Age-related lung diseases, Cellular senescence, Hypoxia-inducible factor (HIF), Longevity assurance genes, Inflammation, And oxidative stress

## Abstract

Cellular senescence and hypoxia-inducible factor (HIF) signaling are crucial in pulmonary aging and age-related lung diseases such as chronic obstructive pulmonary disease idiopathic pulmonary fibrosis and lung cancer. HIF plays a pivotal role in cellular adaptation to hypoxia, regulating processes like angiogenesis, metabolism, and inflammation. Meanwhile, cellular senescence leads to irreversible cell cycle arrest, triggering the senescence-associated secretory phenotype which contributes to chronic inflammation, tissue remodeling, and fibrosis. Dysregulation of these pathways accelerates lung aging and disease progression by promoting oxidative stress, mitochondrial dysfunction, and epigenetic alterations. Recent studies indicate that HIF and senescence interact at multiple levels, where HIF can both induce and suppress senescence, depending on cellular conditions. While transient HIF activation supports tissue repair and stress resistance, chronic dysregulation exacerbates pulmonary pathologies. Furthermore, emerging evidence suggests that targeting HIF and senescence pathways could offer new therapeutic strategies to mitigate age-related lung diseases. This review explores the intricate crosstalk between these mechanisms, shedding light on how their interplay influences pulmonary aging and disease progression. Additionally, we discuss potential interventions, including senolytic therapies and HIF modulators, that could enhance lung health and longevity.

## Introduction

Age-related lung diseases, including chronic obstructive pulmonary disease (COPD), idiopathic pulmonary fibrosis (IPF), and lung cancer, represent major causes of disability and death worldwide. Both these conditions are tightly associated with cellular senescence and hypoxia-inducible factor (HIF) signaling, vital processes of aging lung physiology and pathology (Feng et al. 2024). Cellular senescence, a permanent cell cycle arrest induced by various stressors, including oxidative damage, telomere attrition, and genotoxic insults, plays a major role in aging-related decline (Chei et al. 2024). The phenotype of senescent cells is unique, expressed as the secretion of proinflammatory cytokines, chemokines, and proteases called the senescence-associated secretory phenotype (SASP) (López-Lluch and Rattan 2015). HIF is a key regulator of cellular adaptation to hypoxia, orchestrating multiple processes such as angiogenesis, erythropoiesis, and metabolic regulation. However, its dysregulation contributes to pathological features of pulmonary aging and disease progression. Understanding the interplay between HIF signaling and senescence is crucial for delineating age-related changes in lung function and identifying potential therapeutic targets. Among these, sirtuins and FOXO transcription factors are conserved regulators that enhance stress resistance, metabolic homeostasis, and genomic stability. As aging progresses, impairments in these longevity pathways exacerbated by DNA damage, mitochondrial dysfunction, and epigenetic alterations contribute to the onset of pulmonary diseases. Furthermore, disruptions in hormonal and epigenetic regulation accelerate age-associated pulmonary decline, linking systemic aging mechanisms with lung pathophysiology. Aging leads to a progressive decline in cellular repair mechanisms, antioxidant defenses, and proteostasis, impairing lung function over time(Fu et al. 2024; Gupta et al. 2025). Chronic inflammation, DNA mutations, and metabolic dysregulation drive pathological aging in the lungs, whereas protective adaptations such as autophagy activation may mitigate disease progression.

### Significance of hypoxia-inducible factor (HIF) in lung physiology and pathology

Hypoxia-inducible factor (HIF) is a key transcriptional mediator of cellular responses to low oxygen, which regulates lung physiology and pathogenesis. It is a central regulator of hypoxic adaptation in lung tissues and plays a dual role in maintaining homeostasis and driving pathological processes. (Rattan et al. 2009; Dunham-Snary et al. 2017). At low levels, hypoxia acts as a hormetin, triggering adaptive cellular responses that enhance stress resistance and longevity. This biphasic response, known as hormesis, is a well-established concept in aging research (Rattan 2024). Hypoxia-induced activation of HIF follows a biphasic response, where transient, mild hypoxia can enhance cellular repair mechanisms, improve mitochondrial function, and mitigate oxidative damage. This aligns with the concept of hormesis, a well-established phenomenon in aging biology (Rattan 2022; Calabrese et al. 2024).

However, excessive or prolonged HIF activation skews this adaptive response, fostering fibrosis, inflammation, and disease progression (Rattan 2015). During normal aging, HIF maintains oxygen homeostasis, regulates mitochondrial activity, and supports adaptive stress responses in lung tissues. With advancing age, HIF signaling efficiency declines, leading to reduced stress tolerance and impaired repair mechanisms in lung cells. Chronic HIF dysregulation in aging lungs has been linked to increased oxidative stress, senescence induction, and pro-inflammatory signalling. In the lungs, HIF is also essential for oxygen homeostasis and adaptation to hypoxic environments. Beyond its role in oxygen sensing, HIF modulates cellular metabolism, inflammation, and senescence pathways, directly influencing lung aging (Banoth and Cassel 2018). Under normoxia, HIF is rapidly degraded via proteasomal pathways. However, during hypoxia, a lack of oxygen and inhibition factors will stabilize the concentration of HIF in the cytoplasm. Eventually, it translocates into the nucleus to target gene transcription (Galy et al. 2024). It is involved in the regulation of pulmonary vascular tone and inflammation (Lucero García Rojas et al. 2021).

On the other hand, dysregulation of HIF signaling has been associated with the development and progression of many lung diseases (Li et al. 2021). HIF activation in these diseases may involve structural alterations, including emphysema and pulmonary hypertension observed in COPD. In addition, HIF has been previously linked to pulmonary fibrosis. It may increase the expression of extracellular matrix proteins and inflammatory cell infiltration, contributing to tissue scaring that leads to progressive lung fibrosis and impaired gas exchange. Therapeutic modulation of HIF signaling could provide novel strategies to mitigate pulmonary aging and associated diseases (Martin et al. 2024).

### Relevance of cellular senescence in age-related lung diseases

Cellular senescence, characterized by permanent cell cycle arrest with unique phenotypic changes, has been implicated in the pathogenesis of age-related lung diseases (Yanagi et al. 2017). Accumulation of senescent cells leads to chronic SASP secretion, promoting sustained inflammation, extracellular matrix remodeling, and progressive tissue fibrosis in age-related lung diseases. In the case of idiopathic pulmonary fibrosis (IPF), it has been suggested that senescent cells in this setting may similarly overproduce extracellular matrix proteins and recruit inflammatory cells into the lungs, thereby promoting progressive scarring of the lung and loss of effective gas exchange (Atzeni et al. 2018). Senescence-associated inflammation and SASP factors have been shown to create a tumor-promoting microenvironment, facilitating lung cancer progression and therapy resistance (Koons et al. 2020). HIF not only influences lung disease progression but also modulates the early stages of cellular senescence in normal lung aging. In young cells, HIF supports transient stress adaptation, but in aging cells, its prolonged activation contributes to SASP secretion and chronic inflammation. Understanding the roles of cellular senescence and the SASP in age‐related lung diseases will help establish targeted therapeutic approaches, including eliminating senescent cells. (Shi et al. 2007). Here, we review the contributions of HIF and senescence to lung pathologies, speculate on possible interconnections between these two phenomena, and highlight opportunities for collaborative therapeutic targeting in isolation or combination.

### Hypoxia-Inducible *Factor* (HIF)

#### Molecular structure and isoforms of HIF

The hypoxia-inducible factors are heterodimeric transcription factors that consist of an alpha (α) and a beta (β) subunit. All three HIF-α subunits (HIF-1α, H IF -2α, and the recently discovered HIF -3 α) are oxygen-sensitive and mediate the response of cells to hypoxia by binding to constitutively-expressed ARNT (called additionally a Hif β) (Albadari et al. 2019). HIF-α subunits are structurally characterized by several important domains: an N-terminal basic helix-loop-helix (bHLH) domain for DNA binding, two Per-Arnt-Sim (PAS) domains necessary for heterodimerization with HIF-β subunit and oxygen-dependent degradation domain (ODDD), which in normoxic conditions stabilizes the protein (Albogami et al. 2022). In addition, the c-terminal transactivation domains (TADs) lead to target gene transcriptional activation (Catrina and Zheng 2021). Oxygen sensitivity of HIF-α subunits is regulated via prolyl hydroxylase domain (PHD) enzymes that hydroxylate distinct regulatory proline residues, which target these sequences for ubiquitination and degradation by the proteasome under normoxia. Among isoforms of HIF, HIF-1α and HIF-2α were the most studied (Chen et al. 2022). These two subunits have their unique function but partially overlap each other. HIF-1α is expressed in virtually all cells and quickly responds to hypoxia, controlling the genes too glycolysis, angiogenesis, and erythropoiesis (Cowman and Koh 2022). HIF-2α is more tissue-specific even when broadly expressed, as it is specialized in vascular endothelial cells and some cancers or developmental processes (Dehne et al. 2013). The least well-studied of the isoforms, HIF-3α has no C-terminal TAD, making this a candidate mechanism for acting in opposing fashion to HIF-1α and HIF-2α by forming inactive heterodimers with Arnt sacrificing both activities at sites it binds as a competitive inhibitor in addition (Deshmukh et al. 2012).

#### Mechanisms of HIF regulation

Hypoxia-inducible factors (HIFs) are important transcriptional factors that sense the oxygen concentration change in cellular surroundings (Dzhalilova and Makarova 2022). HIFs activate the transcription of genes involved in angiogenesis, metabolism, and adaptation to hypoxic stress. HIF activity is regulated by mechanisms involving oxygen-dependent and -independent causes (Fedotova et al. 2023).

#### Oxygen-dependent regulation

HIF-1α, a crucial subunit of HIF, is quickly degraded by the oxygen-dependent pathway under normoxic (normal oxygen) circumstances. PHD-mediated regulation is conducted via prolyl hydroxylase domain-containing enzymes that hydroxylate specific proline residues on HIF-1α (Freeman and Barone 2005). The hydroxylation of HIF-1α leads to marking by the VHL protein, which acts like an E3 ubiquitin-protein ligase. Under normal oxygen tension, VHL facilitates the ubiquitination and proteasomal degradation of HIF-1α so that it does not accumulate to be active (Gunton 2020) as the PHD enzyme activity is inhibited by lack of oxygen (required as a co-factor for their enzymatic function.) under hypoxic (low oxygen) conditions (Haddad and Harb 2005). Therefore, HIF-1α is not hydroxylated and manages to evade VHL-mediated degradation (Hu et al. 2016). The stabilized HIF-1α enters the nucleus where it forms a complex with the constitutively expressed aryl hydrocarbon translocator (HIF-1β) and binds to hypoxia-response elements (HREs) in promoter regions responsive genes, initiating transcription of these genes, signaling for cellular adaptation to a hypoxic environment (Hua and Dias 2016).

#### Oxygen-independent regulation

**Fig. 1:**
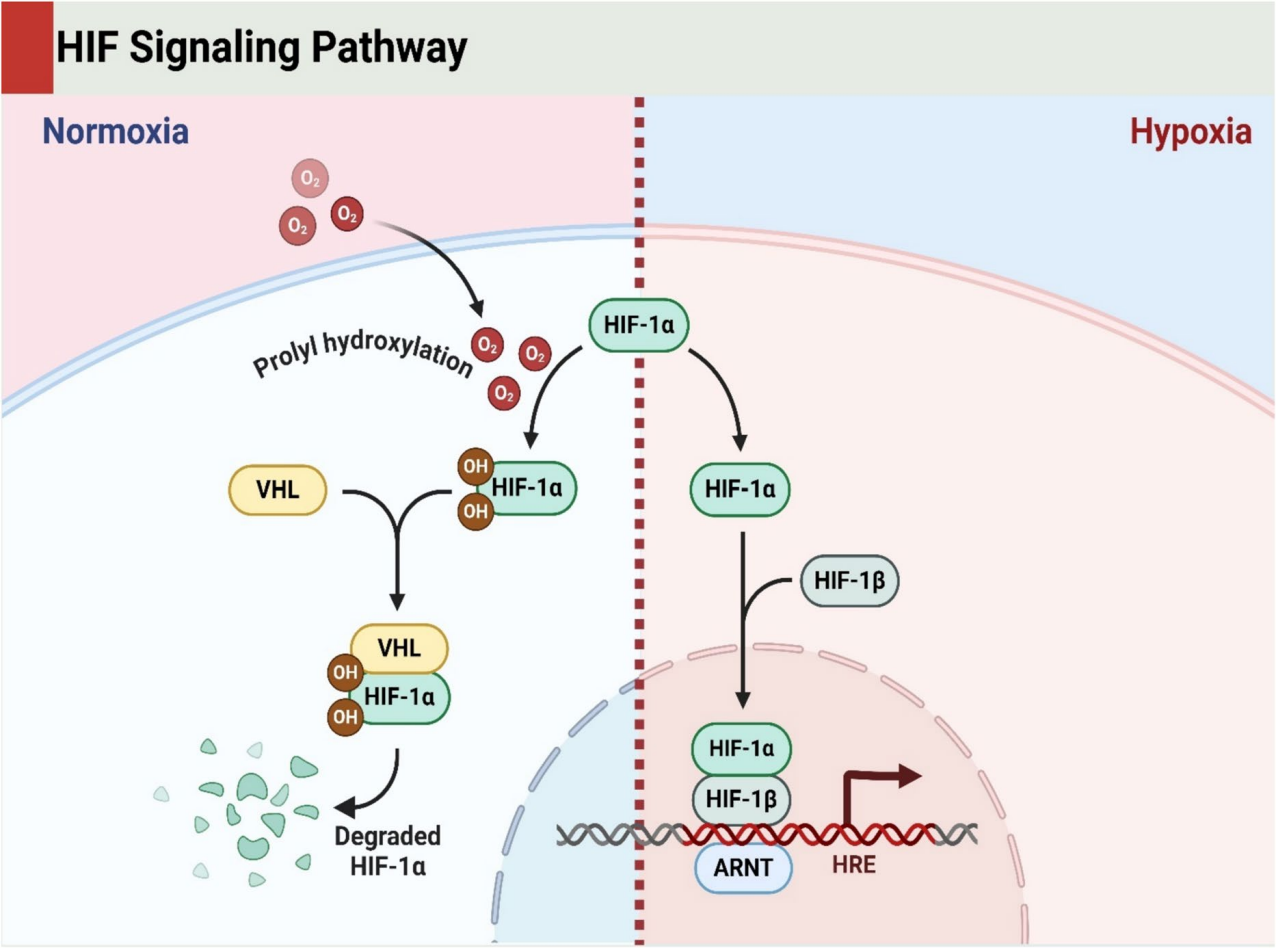
The image illustrates the Hypoxia-Inducible Factor (HIF) signaling pathway. In normoxia, HIF-1α undergoes prolyl hydroxylation, leading to its binding with VHL, resulting in its degradation. In hypoxia, HIF-1α stabilizes, translocates to the nucleus, and dimerizes with HIF-1β. This complex binds to the Hypoxia Response Element (HRE) on DNA, activating gene transcription

Oxygen-independent mechanisms contribute to the modulation of HIF activity, ensuring that cells will respond to hypoxic conditions when the dependence mechanism-related cells are not available (Huang et al. 2017). Overall, a number of factors can affect HIF stability and activity in an oxygen-independent fashion. For example, under hypoxia, ROS could be produced to stabilize HIF-1α by inhibiting PHD function. Furthermore, growth factors such as insulin and EGF will stimulate the PI3K/Akt and MAPK signaling channels, which increase HIF-1α stabilization and action (Koivunen and Kietzmann 2018). In addition, HIF-1α stability and transcriptional activity are further regulated by post-translational modifications, including acetylation, phosphorylation, and SUMOylation (Lee et al. 2004). For instance, acetyltransferase p300/CBP is able to support HIF-1α transcriptional activity though not only promoting strong interaction with co-activators but also loading of the transcriptional machinery. In contrast, factor-inhibiting HIF (FIH) depletes the asparagine residue of HIF-1α by hydroxylation and thereby blocks the interaction with co-activators and represses transcriptional activity of HIF-1 (Loboda et al. 2010). The regulation of HIF is highly complex and includes a combination of oxygen-dependent mechanisms and further-oxygen-independent signaling, enabling cells to adapt optimally to changes in the levels of oxygen or other environmental stimulants (Majmundar et al. 2010). These regulatory pathways are of fundamental interest and also reveal possible elements that can be exploited as potential therapeutics in many conditions involving hypoxia, including cancer or cardiovascular disorders (Manresa and Taylor 2017) (Fig. 1).

#### Physiological roles of HIF in lung development, homeostasis, and responses to hypoxia

The hypoxia-inducible factors, HIFs, are critical for lung development, homeostasis, and adaptive response to hypoxic stress. HIFs are also required during lung development for correct vascularization and alveolarization. HIF-1α and HIF-2α induce the expression of vascular endothelial growth factor (VEGF) that is crucial in the angiogenic process to mediate sufficient blood supply and oxygen diffusion into developing lung tissues (Packer 2021). The vascular development to form functional alveoli, the gas-exchanging units of the lung is required. In lung homeostasis, HIF proteins contribute to the fine-tuning of oxygen supply and metabolic demand (Pawlus and Hu 2013). HIF-1α activates key genes that promote glycolysis, which allows the cells to generate ATP without using oxygen-dependent metabolic pathways. This metabolic flexibility is key for the normal operation of lung tissues, which are accustomed to changes in oxygen exposure (Prabhakar et al. 2020). HIFs are also critical in the hypoxic response of the lung, a situation irregulated in different lung diseases, including chronic obstructive pulmonary disease (COPD), sleep apnea, and pulmonary hypertension (Satija et al. 2021). Under hypoxic conditions, stabilized HIF-1α and HIF-2α status activate transcriptional programs that include processes for erythropoiesis, angiogenesis, and pulmonary vasoconstriction. in pathologies, on the other hand, chronic HIF activation may be involved in diseased states (Schödel et al. 2016). Sustained hypoxia and activation of HIF can cause maladaptive changes in the lung vasculature, which leads to pulmonary hypertension, among other complications (Semenza 2001).

#### Cellular senescence in age-related lung diseases

Cellular senescence is characterized by a state of permanent cell cycle arrest cells are induced to undergo in response to different kinds of stress signals such as DNA damage, oxidative stress, or telomere shortening (Aghali et al. 2022). This is a contributor to the pathogenesis and progression of age-related lung diseases, particularly in disease senescent cell cells (Barnes et al. 2019). During lung aging, the progressive accumulation of senescent cells disturbs tissue homeostasis and can alter repair mechanisms as well as induce chronic inflammation processes that are recognized hallmarks in multiple pulmonary disorders (Cho and Stout-Delgado 2020). One example is chronic obstructive pulmonary disease (COPD), where the lungs contain higher levels of senescent cells. These cell types secrete a range of pro-inflammatory cytokines, chemokines, and proteases together referred to as the senescence-associated secretory phenotype (SASP) (Kirkland and Tchkonia 2020). The SASP perpetuates the pro-inflammatory environment, resulting in tissue damage and fibrosis with impaired lung function. Furthermore, it is possible that in COPD, senescent cells abrogate the regenerative potential of lung epithelial cells, contributing to disease exacerbation (Moss et al. 2022). Another age-related lung disease where cellular senescence has been implicated is idiopathic pulmonary fibrosis (IPF). Senescent fibroblasts and senescent lung epithelial cells are committee producers of factors such as transforming maturation factor beta (TGF-β) that boost fibrosis (Sun et al. 2016). These senescent cells found in IPF lungs are responsible for the overproduction of extracellular matrix components, which causes lung tissue to become stiff and scarred (Barnes 2016). By forming scar tissue, this fibrosis hampers sufficient gas exchange and increasingly limits lung capacity (Ebert et al. 2020).

Another age-related disease conventionally not thought of as such, asthma demonstrates growing proof of cellular senescence in patients aged 65 and older (Hernandez-Gonzalez et al. 2021). Senescent cells greatly contribute to exacerbated inflammation and airway remodeling in asthmatic lungs, resulting in more severe disease that is less treatable in older patients (Kellogg et al. 2021). Cellular senescence also contributes to the development of lung cancer. Through the SASP, senescent cells can establish a tumor-promoting microenvironment and contribute to malignancy transformation and tumorigenesis (Otoupalova et al. 2020). The chronic inflammatory milieu created by senescent cells supports the survival, proliferation, and metastasis of cancer cells. Indeed, the concept of targeting senescent cells as a potential therapeutic approach for age-related lung diseases is consolidated (Phan et al. 2021). Preclinical studies have shown that senolytic drug compounds that clear out senescent cells might be able to reduce fibrosis and improve lung function, as well as dampen down inflammation. Another approach is to try and moderate the SASP so as to reduce its deleterious effects while not actually actively eliminating the senescent cells (Voynow and Shinbashi 2021).

#### Interactions between HIF and cellular senescence

##### Regulation of HIF by senescence pathways

The interaction of hypoxia-inducible factors HIFs and cellular senescence is also essential in relation to age-associated lung diseases (Gao et al. 2023). HIFs are a family of transcription factors that orchestrate cellular hypoxic responses manifested by increase expression in hundreds of genes. Among those, they mostly promote those two equally orthogonal isoforms: HIF-1α and HIF-2 α (Chen et al. 2015). The availability of oxygen tightly regulates all of these parameters and drives key physiologic processes, including cellular metabolism, angiogenesis, and survival. HIFs are deeply involved in the promotion and suppression of senescence pathways irrespective of various theories for cellular senescence, significantly contributing to deteriorating lung diseases among aged populations. Cellular senescence can be triggered by a multitude of stressors, such as oxidant injury, genotoxic insults, and telomere shortening (Oliveira et al. 2022). These stressors engage signaling pathways that ultimately converge on HIF regulation. Similarly, one of the hallmarks of senescent cells, oxidative stress mediates the stabilization of HIF-1α even in normoxic conditions such as ROS8 12 (Poulose and Raju 2014). ROS can inhibit prolyl hydroxylases (PHDs), which in turn are responsible for HIF-1α degradation, resulting in the accumulation of this transcription factor (Salminen 2022).

Additionally, HIF-1α is reported to repress or activate the expression of genes in connection with glycolysis and Angiogenesis. The latter two processes are also believed to be involved in metabolic reprogramming known from senescent cells. Additionally, DNA damage response (DDR) pathways also cross-talk with HIF signaling (Salminen et al. 2016). Importantly, DDR can lead to the activation of ataxia-telangiectasia mutated (ATM) kinase, which in turn activates HIF-1α signaling by augmenting its stability and activity. Overall, this HIF-1α stabilization under DNA damage expands cellular stress adaptations by allowing cells to cope with stress to maintain cell viability as well as preserve senescence-associated secretory phenotype (SASP) properties (Troise et al. 2023). To extend on further example from the hallmarks of cellular aging and senescence, telomere shortening affects HIF activity as well (Chandel and Budinger 2007). Shortened telomeres activate p53 and its target gene p21, initiating the cell cycle arrest and, subsequently, cell senescence (Hwang and Lee 2011). Significantly, p53 also inhibits HIF-1α directly at the level of transcription, so there may be a feedback loop wherein senescence markers can influence HIF activity. The downregulation of HIF-1α by p53 also emphasizes this complex regulatory framework, which integrates cellular senescence and hypoxic stimuli (Mathai et al. 2020). Thus, the regulation of HIFs by senescence pathways offers important new insights into age-dependent pathophysiological processes in the lung. These findings suggested novel therapeutic interventions designed to reduce the burden of chronic lung diseases, repair tissues, and improve lung function with higher human population age through HIF-senescence axis (Renassia and Peyssonnaux 2019).

##### Therapeutic implications and strategies

### Targeting HIF signaling for lung disease intervention

Lung disease intervention can be achieved through the targeting of hypoxia-inducible factor (HIF) signaling. HIF is a master controller of cellular adaptation to conditions of low oxygen tension, inducing the expression of genes that play critical roles in angiogenesis, metabolism, and cell survival (Dawod et al. 2020). Dysregulated HIF signaling in lung diseases such as chronic obstructive pulmonary disease and pulmonary hypertension drives inflammation, fibrosis, and vascular remodeling, enhancing disease progression. A critical consideration in HIF-targeted therapy is the balance between its protective and detrimental effects. The hormetic response of hypoxia suggests that controlled modulation of HIF could serve as a strategy to harness its protective functions while avoiding pathological consequences (Calabrese et al. 2024). Future therapeutic interventions should explore optimal hypoxia dosing strategies to enhance resilience against age-related lung diseases. (Jeganathan and Sathananthan 2020).These include small molecule inhibitors, gene therapy, and oxygen delivery approaches that are focused on restoring normal HIF signaling in order to treat lung disease symptoms (Wijsenbeek et al. 2022). He et al. investigated the expression and regulation of HIF-1α, HIF-2α, HIF-3α, and VEGF in heart and lung tissues from Tibetan sheep, particularly focusing on how those proteins are directly expressed as genes at different ages. Interestingly, HIF-1α and HIF-2α were higher in the lungs of young sheep, whereas HIF-3α and VEGF were elevated in the hearts of old sheep. This research enhances the significance of these factors in acquiescing to hypoxia as and probable method responsible for chronic mountain sickness syndrome among Tibetans (He et al. 2019). Radiofrequency ablation (RFA) is best described as a minimally invasive procedure used to destroy abnormal tissue, most often tumors or arrhythmogenic heart tissue, by applying heat to the tissues that are generated from radiofrequency energy (Widmann et al. 2009; Bhat et al. 2023a). Wan et al. evaluate the prognostic effect of HIF-1α expression on lung cancer patients who accepted radiofrequency ablation (RFA). Elevated HIF-1α levels weakly showed significantly worse overall survival and were established as an independent risk factor for death. These results indicate that HIF-1α may be a useful therapeutic target, which could enhance the efficacy of RFA treatment in lung cancer patients. This study also examines which cellular senescence plays in cancer prognosis (Wan et al. 2020). GDC is a multienzyme complex responsible for the catabolism of glycine, with subsequent release of CO2 and NH3 and the formation of a methylene group. It is highly involved in cellular respiration and photorespiration of plants (Schulze et al. 2016). Berezowska et al. analyze the level of GLDC and HIF-1α in early-stage non-small cell lung cancers (NSCLC). In both instances, low expression of these was discovered to be a negative prognostic factor for better survival and was significantly predictive of worse outcomes. Together, these results suggest that targeting these pathways might be beneficial for NSCLC therapy. The study also aimed to explore the role of cellular senescence in lung cancer development (Berezowska et al. 2017b).

**Fig. 2:**
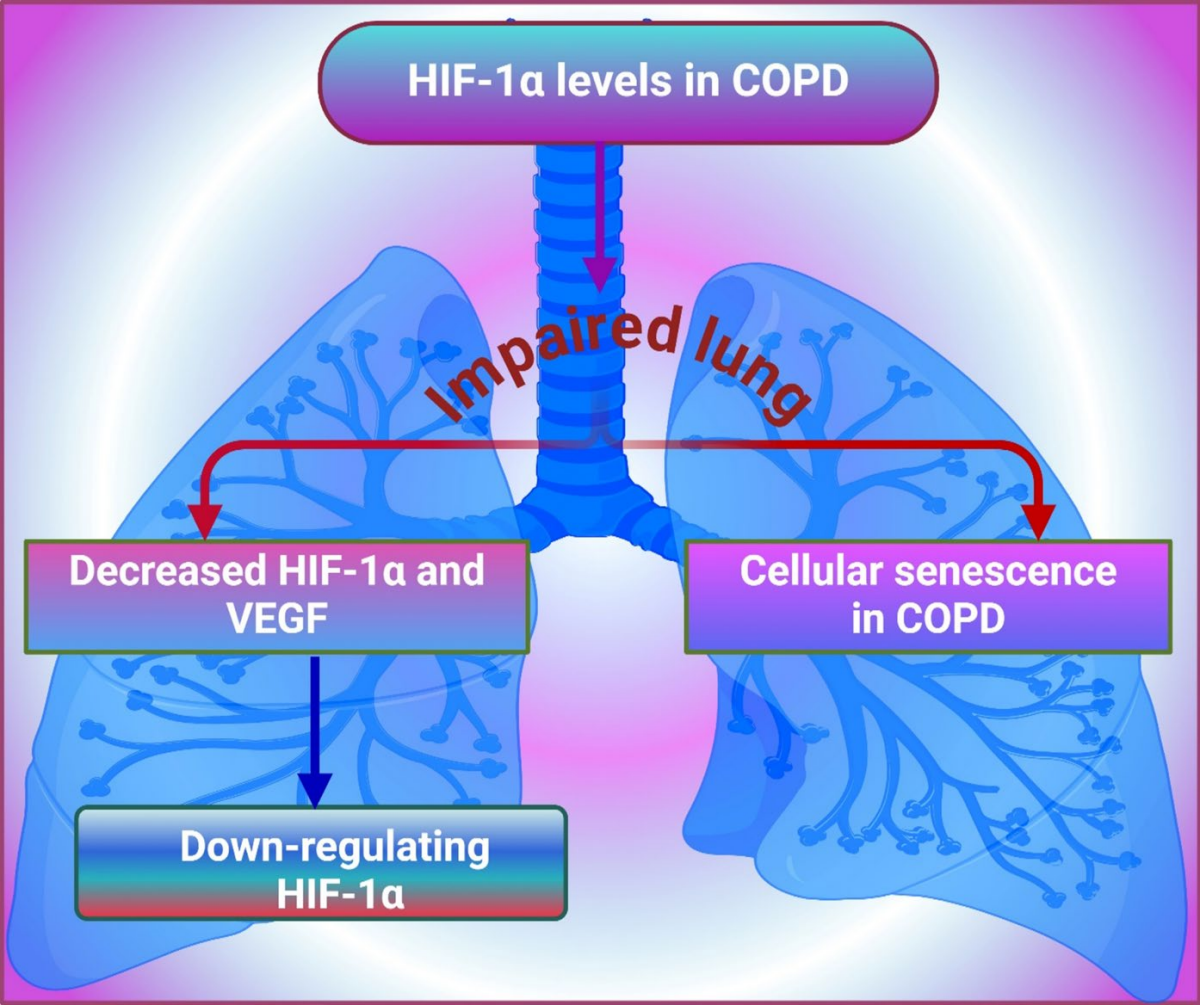
The image illustrates the protein levels of HIF-1α in lung effigies from COPD/emphysema patients. It shows that impaired lung maintenance is indicated by significant decreases in HIF-1α and VEGF proteins in severe to very severe COPD. These findings suggest that down-regulating HIF-1α could aid clinical therapy for COPD. Additionally, the relevance of cellular senescence in COPD progression is highlighted

Hypoxia or oxygen promotes VEGF (vascular endothelial growth factor) expression, and hyperoxia exposure to high levels of that gas leads to impact. The most direct consequence of this action is a downregulation of VEGF, which inhibits angiogenesis and results in conditions such as retinopathy and an impaired wound-healing response (Liu et al. 1995). Hosford et al. clarify the effects of hyperoxia on VEGF, its receptors, and HIF-2α in newborn rat lungs. The studies show that hyperoxia suppressed the normally occurring developmental upregulation of HIF-2α and VEGF, both essential for angiogenesis and alveolarization. They speculate that such suppression might reduce lung development and will assume that our findings lead to the identification of potential targets for intervention in hyperoxia-induced injury to the developing lung. Additionally, this research addresses the consequences of cellular senescence in lung formation. (Hosford and Olson 2003). Progressive lung diseases, COPD and emphysema are conditions causing the difficulty of breathing. Smoking is the leading cause that damages airways and air sacs within the lungs, resulting in too little airflow (Christenson et al. 2022). Yasuo et al. determine the protein levels of HIF-1α features in lung effigies from COPD/emphysema patients. Their results showed that at the molecular level, impaired lung maintenance is already indicated by significant decreases in HIF-1α and VEGF proteins in severe to very severe COPD. These results suggested that down-regulating HIF-1α could be helpful for clinical therapy of COPD. The last point of interest is the relevance of cellular senescence within the COPD course (Yasuo et al. 2011) (Fig. 2).

EphB4 and HIF-1α play a vital role in lung cancer by promoting tumor growth, angiogenesis, and metastasis. This means that they can act on each other to help the cancer cells progress and are promising targets for treatment (Bhat et al. 2023b; Zhi et al. 2024). Zhu et al. detected the expression level of EphB4 and HIF-1α in lung cancer tissue. This suggests that both proteins are highly expressed in lung cancer tissues and regulated by the malignant state of lung cancers. This study illuminates the perspectives of the clinical potential of targeting these pathways for better outcomes in lung cancer therapy (Zhu et al. 2005). Lung vascular development is the formation of a complex blood vessel network, which is necessary for gas exchange. This process is linked to vasculogenesis, angiogenesis, and vessel maturation, which are governed through signaling pathways (Udan et al. 2013). Asikainen et al. Examines the influence of hypoxia-inducible factors (HIFs) on lung vascular development in preterm baboons. In summary, the study shows that stimulation of HIF by PHD inhibition increased lung angiogenesis and may be an approach to treat BPD. The findings indicate HIF pathways could be targeted to enhance lung development in preemies, taking into account cellular senescence (Asikainen et al. 2006) (Table 1).

**Table 1:** This table highlights how HIF signaling shifts from adaptive to dysregulated states, contributing to age-related lung dysfunction and disease onset. Additionally, it outlines therapeutic insights targeting HIF pathways to mitigate senescence-driven lung impairments and improve aging-related lung health

Study Model	Aging Stage	HIF-Senescence Interactions	Pathological Implications	Therapeutic Insights	Reference
Tibetan Sheep	Young → Old	HIF-1α & HIF-2α active in young lungs, HIF-3α increases with age	Shift from adaptation to chronic hypoxia	Role in chronic mountain sickness, potential HIF modulation	(He et al. 2019)
Lung Cancer Patients	Middle → Old	High HIF-1α accelerates senescence	Poor prognosis, therapy resistance	Targeting HIF-1α for prognosis improvement	(Wan et al. 2020)
NSCLC Patients	Middle → Old	GLDC and HIF-1α contribute to metabolic changes	Increased cancer progression	Dual targeting of GLDC and HIF-1α	(Berezowska et al. 2017a)
Newborn Rats	Neonatal	Hyperoxia suppresses HIF-2α and VEGF	Arrested lung development, senescence initiation	Potential intervention for neonatal lung injury	(Hosford and Olson 2003)
COPD/Emphysema Patients	Old	Decreased HIF-1α and VEGF accelerate lung senescence	Worsening COPD, impaired lung regeneration	HIF-1α modulation to prevent COPD progression	(Yasuo et al. 2011)
Preterm Baboons	Neonatal → Young	HIF stimulation enhances angiogenesis, but imbalance affects lung development	Increased risk of bronchopulmonary dysplasia (BPD)	HIF pathway-based lung development therapies	(Asikainen et al. 2006)
Preterm Baboons	Neonatal → Young	Downregulation of HIF-1α and HIF-2α	Lung hypoplasia in preterm neonates	HIF-targeted intervention in preterm care	(Asikainen et al. 2005)

### Modulating cellular senescence and SASP in age-related lung diseases

Interventions targeting cellular senescence and the SASP have shown promise in addressing age-related lung diseases (Barnes 2019). Cellular senescence-derived SASP contributes to lung pathologies, such as chronic obstructive pulmonary disease (COPD) and idiopathic pulmonary fibrosis (IPF), with inflammation and tissue remodeling (Kirkland and Tchkonia 2020). The detrimental effects can be alleviated by targeting senescent cells or modulating SASP (Parimon et al. 2023). These strategies incorporate senolytics that eliminate its cells preferentially and senomorphics that turn off SASP without killing the cell. By targeting the processes, including inflammation, lung function repair, and disease progression, these strategies would be advantageous for the therapy of age-related lung diseases (Wan et al. 2023). Petruccelli et al. explored multiple molecular pathways of the lung after the aging process, mainly on HIF, VEGF, p53, and several other markers. Increased expressions of pro-apoptotic markers and VEGF in aged rat lungs demonstrate a balance between apoptosis and survival mechanisms. Taken together, the results revealed that inhibition of these pathways can reduce age-specific decline in lung function and cellular senescence (Petruccelli et al. 2010). Adrenomedullin is a peptide hormone that functions in numerous vital physiological processes such as vasodilation, angiogenesis and controlling blood pressure. Because of the crucial function it has in cardiovascular homeostasis as well as its involvement in pathology, such as heart failure, hypertension, and cancer (Martínez-Herrero and Martínez 2022). Hwang et al. investigated age-related changes in AM and HIF-1 expression and activity in rat lungs under hypoxia. The reduced hypoxic response observed in older-experimented rats indicates an age-related dysfunction of the HIF-AM pathway. Notably, this illustrates the promise of therapies targeting HIF signaling in combating age-related lung functional loss and cellular senescence (Hwang et al. 2007). The oxygen-sensing system of organisms is based on the determination of fluctuations in the level of molecular or cellular oxygen by hypoxia-inducible factors (HIFs). This allows for the regulation of gene expression, making it possible to adjust cellular metabolism, angiogenesis, or even cell fate in order to accommodate low oxygen bioavailability (Lee et al. 2020). Kirschner et al. study the adaptation of this oxygen-sensing system in lung development by examining HIF-regulated genes. Their findings indicated that the HIF-PHD system changes from intrauterine to neonatal phases, underscoring its importance during lung development and its candidacy as a target for treatment of prematurity-fueled lung diseases. The current study also investigates the participation of cellular senescence in this process of adaptation (Alharbi et al. 2021; Kirschner et al. 2022).

Resected lung cancers, meaning tumors that have been cut out of the lung, are usually sent for histopathological analysis. In many cases, this procedure is used to treat non-small cell lung cancer and small cell lung cancer. The goal of surgical resection is to remove all cancerous tissue with the intent to cure or prolong life (Ghamati et al. 2021; Bhat et al. 2024). This and the surgery type, which will depend on the tumor size, location, and patient health, may go from lobectomy to pneumonectomy. After surgery, patients often need further treatment like chemotherapy, radiation, or targeted therapies in order to prevent cancer from coming back and treat any other tumor cells (Schabath and Cote 2019; Alharbi et al. 2022). Takasaki et al. examining HIF-1α expression in surgically resected lung cancers and its correlation with tumor proliferation and antiapoptosis. The data indicate that the high expression of HIF-1α was associated with tumor proliferating and poor prognosis. Therefore, the inhibition or knockdown of HIF-1α may abrogate these phenotypes. In addition, the study points to the effect of cellular senescence on lung cancer results (Takasaki et al. 2016). Development of the pulmonary vasculature involves this assembly and maturation process. This process starts early in the embryonic stage and extends into postnatal also (Avdalovic 2015; Bhat et al. 2022). There are various key stages, such as vasculogenesis, where new vessels form blood vessels from endothelial cells, and angiogenesis, during which existing blood vessels branch out to new fields. For adequate gas exchange and, therefore, overall lung function, the right development of the lungs is important (Blanco et al. 2016). The defects or abnormalities in this mechanism lead the way to various pulmonary diseases and problems (Townsley 2012; Chellappan et al. 2022). Rajatapiti et al. determine HIF expression in normal human lung development. This indicates that HIF-2α is essential for the optimal structural development of the pulmonary vasculature and especially in late gestation where it readies the fetus for extrauterine life. This study establishes a baseline for assessing impaired pulmonary development and also raises the possibility of cellular senescence contributing to lung maturity (Rajatapiti et al. 2008). MicroRNA-126, miR-126, is a small non-coding RNA molecule that has been proven to regulate many biological processes with emphasis on angiogenesis and general vasculature functioning (Chen et al. 2021). It works by degrading the translation of some specific mRNAs or actually destroying them (Thapa et al. 2023a; Liao et al. 2024). Among the miRNAs that have been proven to be crucial in the proper functioning and maintenance of vascular homeostasis is miR-126, whose disturbances have been linked with various types of cancer but also cardiovascular complications of diabetes (Pradhan et al. 2019; Nammian et al. 2020). As the previous studies demonstrate miR-126 could be an attractive biomarker for diagnosis of disease and also an effective therapeutic target to modulate angiogenic pathways (Zapilko et al. 2020). Alique et al. studied the Inhibition of microRNA-126 with HIF-1α in endothelial cell senescence. Their results revealed that senescent endothelial cells exhibit impairment of tube formation and wound healing with low HIF-1α expression. The present study indicates that targeting the microRNA − 126/HIF-1α axis may be beneficial for treating age-related vascular diseases and highlights an association between cellular senescence and vascular health (Alique et al. 2019; Rohilla et al. 2023). Fetal lung maturation is the last process of alveolar development as well as production of surfactant which is important for breathing after birth. This process is completed by around 34 weeks of gestation to permit adequate gas exchange (Liggins 1994). Lazic et al. characterizes the impact of prenatal ethanol exposure on fetal lung maturation and immune function. The findings suggest that ethanol exposure impairs lung development as well as immune defense by interfering with the expression of HIF-1α, HIF-2α and VEGF. These data demonstrate the phenotypic consequences of ethanol exposure and suggest possible targets for interventions aimed to minimize adverse impact of prenatal ethanol exposure on lung health and senescence (Lazic et al. 2011; Singh et al. 2024).

**Fig. 3:**
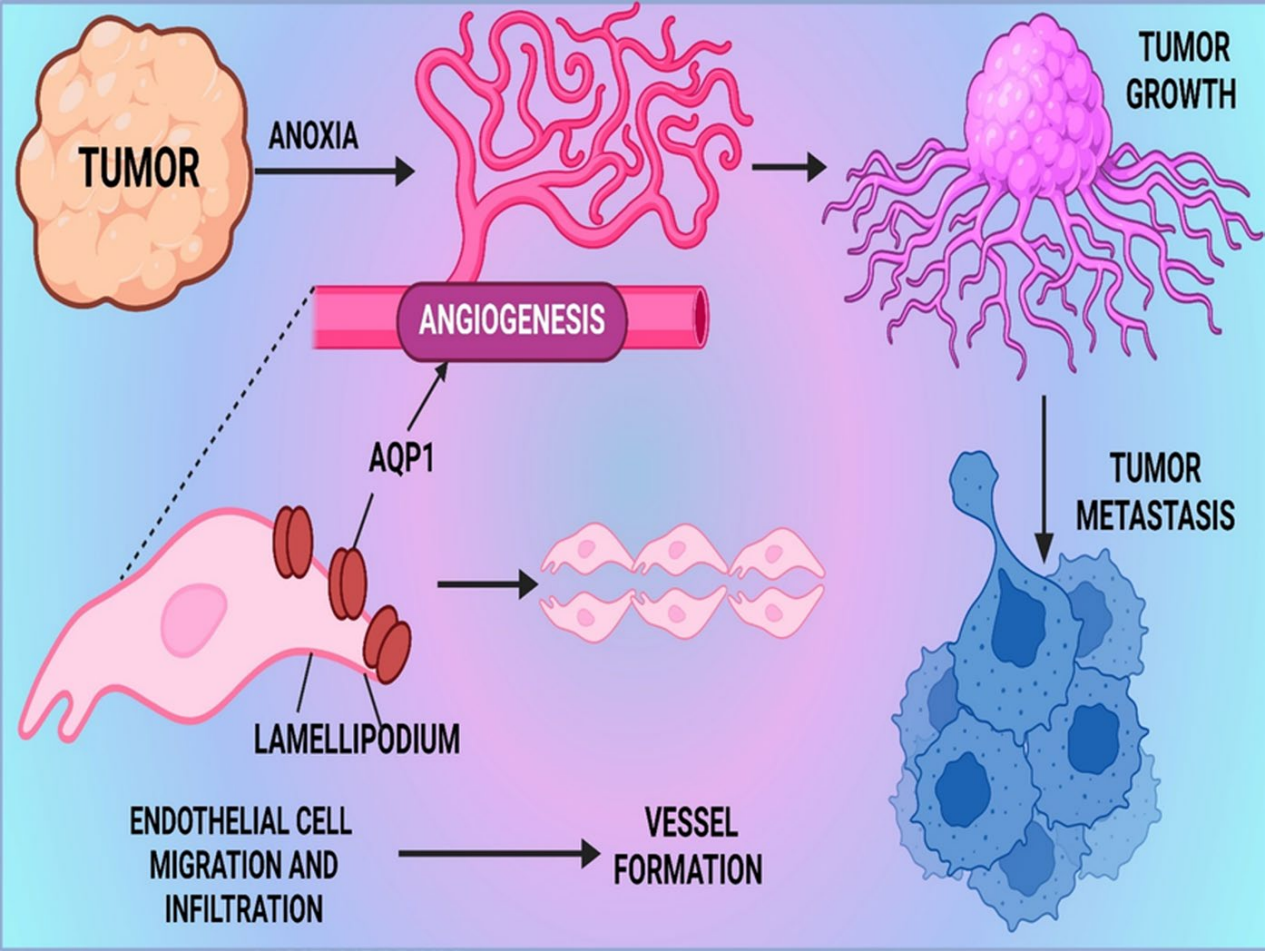
The image depicts the role of AQP1 in tumor growth and metastasis. Anoxia in tumors triggers angiogenesis, leading to tumor growth and metastasis. The findings in a LUNG cancer mouse model indicated that AQP1 deficiency reduces tumor growth and lung metastasis. Interestingly, HIF-1α expression was higher in tumors from AQP1-null mice compared to wild-type. This suggests AQP1 and HIF-1α as potential cancer therapy targets

Moxibustion is a traditional Chinese medicine therapy that consists of burning dried mugwort (moxa) on particular points on the body. Helps to increase circulation, strengthen the immune system, and brings a general state of wellbeing (Deng and Shen 2013). Mao et al. evaluated the influence of moxibustion combined with cisplatin on tumor hypoxia and vascular normalization in mice bearing Lewis’s lung cancer. The demonstrates that the combination therapy reduced HIF-1α and VEGF levels in lung tumors, leading to the reduction of tumor hypoxia and subsequent inhibition of tumour growth. Taken together, these results indicate that HIF signaling has the potential to be an effective therapeutic target with other regimens for enhanced cancer therapy and contribution of cellular senescence (Deng and Shen 2013). Aquaporin 1 (AQP1) is a water channel protein that is present in the membrane of the cell through which it rapidly facilitates the transport of water. It maintains water homeostasis in different types of tissues and organs (Chaumont and Tyerman 2014). Font et al. investigated the AQP1 involved in the growth and metastasis of tumors through a cancer mouse model. The findings clearly indicated that AQP1-deficiency decreases tumor growth and lung metastasis. Nevertheless, HIF-1α expression was higher in tumors from AQP1 null mice compared to wild type. This research showed that AQP1 and HIF-1α could be selected as potential targets for cancer therapy and cellular senescence (Esteva-Font et al. 2014) (Fig. 3; Table 2).

**Table 2:** This table summarizes the role of hypoxia-inducible factors (HIFs) in age-related lung diseases, highlighting age-related changes, senescence implications, and potential therapeutic targets for improving lung health and treatment outcomes

Model	HIFs and Aging	Senescence Implications	Potential Implications	Reference
Aging rat lungs	↑HIF, ↑VEGF, ↑p53 with aging	↑Pro-apoptotic markers, survival mechanisms	Target aging-related lung function decline	(Petruccelli et al. 2010)
Aging rat lungs under hypoxia	↓HIF-1 activity in older rats	Impaired hypoxic response	Address age-related hypoxic response decline	(Hwang et al. 2007)
Lung development in rats	Changes in HIF-PHD during development	Adaptation to hypoxia, senescence role	Treat prematurity-associated lung diseases	(Kirschner et al. 2022)
Human lung cancer tissues	↑HIF-1α correlates with tumor growth	↑Tumor proliferation, poor prognosis	Mitigate tumor growth, improve prognosis	(Takasaki et al. 2016)
Normal human lung development	Crucial role of HIF-2α	Pulmonary vascular development	Evaluate abnormal pulmonary development	(Rajatapiti et al. 2008)
Endothelial cells	↓HIF-1α in senescent cells	Impaired tube formation, wound healing	Benefits of age-related vascular diseases	(Alique et al. 2019)
Prenatal ethanol-exposed lungs	Disrupted HIF-1α, HIF-2α, and VEGF	Impaired lung development and immunity	Mitigate effects of prenatal ethanol exposure	(Lazic et al. 2011)
Lewis lung carcinoma mice	↓HIF-1α and VEGF with therapy	Alleviates tumor hypoxia	Improve cancer treatment outcomes	(Mao et al. 2023)
cancer mouse model	↑HIF-1α in AQP1-deficient tumors	Reduced tumor growth, lung metastasis	Target AQP1 and HIF-1α in cancer therapy	(Esteva-Font et al. 2014)

### Combinatorial approaches targeting HIF and senescence pathways

Combinations of inhibitors that target the HIF (hypoxia-inducible factor) and senescence pathways are promising with regard to new therapeutic interventions in lung diseases. Hypoxia is a hallmark of most forms of chronic lung disease, which activates HIF, resulting in the expression of genes that favor enhanced cell survival, angiogenesis, and metabolic adaptation (Troise et al. 2023). Additionally, cellular senescence promotes inflammation, fibrosis, and tissue repair failure, contributing to lung disease pathogenesis (Saito et al. 2015; Thapa et al. 2023b). Combining these two strategies can provide superior treatment efficacy as multiple facets of lung diseases are addressed by HIF and senescence pathways. For instance, treating with a combination of HIF inhibitors and senolytic compounds that selectively remove aged cells might help stop inflammation/integrity while improving lung function (Li et al. 2017; Parks et al. 2017). For diseases like chronic obstructive pulmonary disease (COPD) and idiopathic pulmonary fibrosis (IPF), this dual strategy offers hope of increasing the efficacy and quality of life in patients with these conditions (Bonham et al. 2020). Li et al. examine how CCR7 can be upregulated in NSCLC by HIF-1α and HIF-2α. These factors’ expression had a strong correlation with lymph node metastasis, so the HIF-CCR7 pathway would be an attractive target for managing NSCLC metastasis. The study also examines the consequences of cellular senescence in this area (Li et al. 2008; Yin et al. 2023).

Pulmonary Sarcoidosis is a chronic inflammatory disease with the formation of granuloma in the lungs. It can cause respiratory symptoms, including cough and shortness of breath, and in rare cases, lead to fibrosis (Belperio et al. 2022). Pulmonary sarcoidosis is a chronic inflammatory disease that forms granulomas in the lungs. Respiratory symptoms, such as a cough and shortness of breath on exertion. In some cases, it can result in fibrosis (Spagnolo et al. 2018; Zhao et al. 2022). Piotrowski et al. studies the HIF-1A/VEGF/ING-4 axis in pulmonary sarcoidosis and correlate an increase in HIF-1A and VEGF with poor lung capacities among patients. These results indicate that this axis could be likely to manage sarcoidosis, especially in inflammation and fibrosis stages. The study further illustrates the importance of cellular senescence in this disease (Piotrowski et al. 2015; Cao et al. 2024).

Non-small cell lung cancer causes about 85% of lung cancer. Subtypes include adenocarcinoma, cancer, squamous cell carcinoma, and different large cell carcinomas (Molina et al. 2008; Chen et al. 2024). Zuo et al. to evaluate HIF-1α and VEGF-C expression in non-small cell lung cancer (NSCLC). Aberrantly increased both in NSCLC, high levels of the two proteins were associated with advanced disease stages and metastasis, so inhibition of them could contribute to better prognosis and treatment results for NSCLC. Secondly, this research also talks about how cellular senescence contributes to lung cancer (Zuo et al. 2008; Lou et al. 2024). Non‑small cell lung cancer of the glandular cells in the individuals is known as lung adenocarcinoma. This is the most common type of lung cancer in non-smokers. Glands and mucin drive this malignant disposition (Zappa and Mousa 2016; Yi-Wen et al. 2018). Jing et al. studied the expression of HIF-1α, COX-2, and E-cadherin in lung adenocarcinoma. HIF-1α was correlated with tumor size, and COX-2 was associated with metastasis and staging. These results indicated that the combination acted in a combinatorial, not synergistic, manner and that the HIF ad COX-2 pathway is a potential point of intervention for lung adenocarcinoma. Ultimately, the study highlights cell senescence’s critical importance across all stages of cancer development (Jing et al. 2012; Lin et al. 2016).

Lung neuroendocrine tumors (NETs) comprise a spectrum of malignancies arising from neuroendocrine cells in the lung. These include low-grade typical carcinoid and high-grade small-cell lung carcinoma, with varying degrees of aggressiveness treatment options (Pelosi et al. 2017; Thapa et al. 2023c). Salvia et al. analyses angiogenesis/hypoxia in Lu-NETs, questioning for the first time if there are differences between right and left lung parenchyma. These results point to distinct angiogenic and hypoxic conditions depending on the location of the tumor, with left tumors showing lower levels of vascularization but a more severe degree of hypoxia. Their study reveals the druggable features of Angiogenesis and Hypoxia pathways for treating Lu-NETs. While taking into account the influence on cellular senescence (Pelosi et al. 2017; Jiang et al. 2024). Bevacizumab, a monoclonal antibody that inhibits VEGF (vascular endothelial growth factor), was also available. It has been indicated in the treatment of many cancers, as it blocks blood vessels that provide tumors with nutrients and oxygen, which will restrict the tumor's growth (Ferrara et al. 2005; Du et al. 2025). Duggan et al. analyze the effects of intravitreal bevacizumab on lung expression of angiogenesis biomarkers in neonatal rats with hypoxia-induced angiogenesis. This study indicates that bevacizumab influences VEGF and HIF-1α quantities, causing unfavorable outcomes in the pulmonary center. They speculate that there may be a risk associated with the systemic effects of local treatments and propose to take cellular senescence into account in therapeutic approaches (Duggan et al. 2021; Zhu et al. 2024).

Respiratory distress syndrome is a life-threatening condition seen most often in premature infants due to an inadequate amount of surfactant within their lungs. It is associated with breathing difficulty and hypoxia, which necessitates immediate medical care (Lou et al. 2021; Bos and Ware 2022). Grover et al. explored HIF-1α and HIF-2α expression in an experimental model of severe RDS in preterm lambs. These findings demonstrate that RDS leads to reduced HIF-1α and VEGF mRNA expression, which may contribute to the long-term pulmonary abnormalities observed. Their work uncovers the potential of targeting HIF pathways to ameliorate RDS in preterm neonates and defines an earlier unknown role for cellular senescence in lung injury (Grover et al. 2007; Hu et al. 2022). Newborn lungs are still developing and have to change over to breathe air. Surfactant production is important to reduce the surface tension and prevent alveoli from collapsing, which helps in proper gas exchange (Nkadi et al. 2009; Thapa et al. 2023d). Barresi et al. study the expression of endoglin (CD105) in human fetal and neonatal lungs. The results indicate that HIF-1 regulates endoglin expression and raises the possibility that induction of this gene may be related to lung vasculogenesis. Their findings indicate that the inhibition of HIF-1 or endoglin, either alone or in combination, could constitute a strategy to alleviate some lung development defects and cellular senescence disorders associated with pathological conditions (Barresi et al. 2008; Li et al. 2024). similarly, Xia et al. explore the expression of HIF-1α in non-small cell lung cancer and also its clinical significance. Since high expression of HIF-1α was correlated with tumor differentiation and stage in NSCLC, HIF-1α might be a better molecular target for improving NSCLC treatment access. Another role in cellular complexion and cancer resistance of HIF-1α (Xia et al. 2004; Rattan 2012; Ruan et al. 2023).

## Conclusion and future perspective

In this review, the roles of HIFs and cellular senescence on age-related lung diseases are highlighted to delineate their reciprocal involvement in these fundamental biological processes. Our key findings demonstrate major roles for HIFs, in particular HIF-1α and HIF-2α, in the lung response to hypoxia, including protective functions to maintain oxygen homeostasis, promoting angiogenesis when possible, and orchestrating metabolic adaptation. Cellular senescence is an established cause of aging and age-related diseases. In the lungs, senescence cells increase through time, and they release a variety of pro-inflammatory cytokines, growth factors, and protease- along termed as from SASP. In contrast, HIFs, through hypoxia induction, can be the driving force for senescence where they accelerate cellular aging. Despite advancements in understanding HIF’s role in lung aging, significant knowledge gaps remain. The extent to which HIF signaling interacts with broader aging mechanisms, such as mitochondrial dysfunction and epigenetic regulation, is still underexplored. Additionally, further investigations should evaluate the threshold at which HIF-driven adaptation transitions into pathology and whether targeted modulation of HIF can extend lung healthspan without promoting disease progression. Moreover, the bidirectional relationship implies that individually targeting either HIF signaling or cellular senescence would fall short of achieving complete remediation in age-related lung diseases.

Investigations in the future should evaluate further the therapeutic ability to target these pathways, prioritizing their crosstalk between them in order to design new strategies that neutralize more efficiently the events of aging undermining lung health. Another key unresolved question is how transient versus chronic HIF activation affects different lung cell populations over time. Identifying biomarkers that distinguish adaptive versus maladaptive HIF responses could aid in developing precise therapeutic interventions for age-related lung diseases. With great potential for enhancing the quality of life in older persons with chronic lung disease, this approach can eventually result in better clinical outcomes as well as increased longevity.

## Data Availability

No datasets were generated or analysed during the current study.
